# Supplementary material for: Atherosclerosis Burdens in Diabetes Mellitus: Assessment by PET Imaging
Source: Int J Mol Sci. 2022 Sep 6;23(18):10268. doi: 10.3390/ijms231810268 (PMC9499611; doi:10.3390/ijms231810268)
Supplement: Supplementary file 1 [file ijms-23-10268-s001.zip › ijms-1873636-supplementary.pdf]

Atherosclerosis Burdens in Diabetes Mellitus: Assessment by PET Imaging

SUPPLEMENTARY MATERIALS

Contents

Search Strings for Literature Retrieval 2

Table S1: Jan 2000 – Mar 2022 Studies on Disease Mechanisms and Targeting 4

Table S2: Jan 2000 – Mar 2022 Studies on Early Detection and Prevalence of Arterial FDG or NaF Uptake 8

Table S3: Jan 2000 – Mar 2022 Studies on Cardiovascular Risk in DM 11

Table S4: Jan 2000 – Mar 2022 Studies on Disease Progression 12

Table S5: Jan 2000 – Mar 2022 Studies on Therapy 13

Full search strategies for Pubmed/Medline, Web of Science, and Embase

(((((Positron emission tomography[Title/Abstract]) OR (PET [Title/Abstract]) OR (PET/CT[Title/Abstract]) OR (PET/MRI[Title/Abstract]))

AND

(((Sodium Fluoride[Title/Abstract]) OR (NaF[Title/Abstract]) OR (18 NAF[Title/Abstract]) OR (NaF-PET[Title/Abstract]) OR (NAF F18[Title/Abstract]) OR (Sodium fluoride F18[Title/Abstract]) OR (NaF-PET-CT[Title/Abstract]) OR (18F-NaF[Title/Abstract]) OR (18-F-NaF[Title/Abstract]) OR (18-F NaF[Title/Abstract]) OR (F-18 NaF[Title/Abstract])))

AND

((diabetes[Title/Abstract]) OR (diabetic[Title/Abstract]) OR (prediabetes[Title/Abstract]) OR (pre-diabetic[Title/Abstract]) OR (iddm[Title/Abstract]) OR (niddm[Title/Abstract]) OR (mody[Title/Abstract]) OR (t1dm[Title/Abstract]) OR (t2dm[Title/Abstract]) OR (t1d[Title/Abstract]) OR (t2d[Title/Abstract]))

AND

(((Atherosclerosis[Title/Abstract]) OR (Artery[Title/Abstract]) OR (stenosis[Title/Abstract]) OR (narrowing[Title/Abstract]) OR (atherosclerotic[Title/Abstract]) OR (Plaque[Title/Abstract]) OR Atheroma[Title/Abstract]) OR (artery disease[Title/Abstract]) OR (cardiovascular[Title/Abstract]) OR (heart[Title/Abstract]) OR (arteries[Title/Abstract]) OR (cardiac[Title/Abstract])))) NOT (Review[Publication Type]) AND (2000:2022[pdat])

Embase

1	PET.mp. or exp positron emission tomography/ 286939	
2	exp positron emission tomography-computed tomography/or exp fluorodeoxyglucose f 18/ or exp positron emission tomography/ 216021	
3	fluorine 18/ or positron emission tomography-computed tomography/ or fluoride sodium/ or fluoride/ or NaF.mp. 113217	
4	Diabetes. mp. or exp diabetes mellitus/ 1309286	
5	Atherosclerosis.mp. or carotid atherosclerosis/ or brain atherosclerosis/ or exp atherosclerosis/ or aortic atherosclerosis/ or coronary artery atherosclerosis/ or experimental atherosclerosis/ 309457	
6	2 or 3	272691
7	1 and 4 and 5 and 6	430
8	limit 7 to yr="2000 -Current"	426
9	exp diabetes mellitus/	1136593
10	8 and 9	377
11	limit 10 to article	142

Scopus

(positron AND emission AND tomography OR pet OR pet/ct OR pet/mri) AND (fluorodeoxyglucose AND f18 OR fluorodeoxyglucose AND f 18 OR fludeoxyglucose AND f18 OR fludeoxyglucose AND f 18 OR fluorine-18-fluorodeoxyglucose OR fluorine-18-fludeoxyglucose OR fluorodeoxyglucose AND 18f OR fluorodeoxyglucose 18 f OR fludeoxyglucose AND 18f OR fludeoxyglucose 18 f OR 18 fdg OR 18fdg OR 18 f AND fdg OR 18f AND fdg OR f18 AND fdg OR f 18 fdg OR fdg AND 18f OR fdg 18 f OR fdg AND f18 OR fdg AND f 18 OR fdg18 OR 18 fluorodeoxyglucose OR 18fluorodeoxyglucose OR 2-fluoro-2-

deoxy-d-glucose OR 2-fluoro-2-deoxyglucose OR 2-fluoro-2-deoxy-glucose OR sodium AND fluoride OR naf OR 18 naf OR naf-pet OR naf AND f18 OR sodium AND fluoride AND f18 OR naf-pet-ct OR 18f-naf OR 18-f-naf OR 18-f AND naf OR f-18 AND naf) AND (diabetes OR diabetic OR prediabetes OR pre-diabetic OR iddm OR niddm OR mody OR t1dm OR t2dm OR t1d OR t2d) AND (atherosclerosis OR artery OR stenosis OR narrowing OR atherosclerotic OR plaque OR atheroma OR artery AND disease OR cardiovascular OR heart OR arteries OR cardiac)

Cochrane library

"Positron emission tomography" OR "PET" OR "PET/CT" OR "PET/MRI" in Title Abstract Keyword AND “sodium fluoride“ OR “naf“ OR “18 naf“ OR “naf pet“ OR “naf pet ct“ OR “18f naf“ OR “18 f naf“ OR “18 f naf“ OR “f 18 naf“ in Title Abstract Keyword AND "diabetes" OR "diabetic" OR "prediabetes" OR "pre-diabetic" OR "iddm" OR "niddm" OR "mody" OR "t1dm" OR "t2dm" OR "t1d" OR "t2d" in Title Abstract Keyword AND “atherosclerosis“ OR “artery“ OR “stenosis“ OR “narrowing“ OR “atherosclerotic“ OR “plaque“ OR “atheroma“ OR “artery disease“ OR “cardiovascular“ OR “heart“ OR “arteries“ OR “cardiac“

Table S1: 2000 – Mar 2022 Studies on Disease Mechanisms and Targeting

First author, time [Ref. #] Site	Study subjects, n (females) Age in years Mean±SD or range	Material	Tracer	Arterial segment	Purpose	Quantification	Main findings	Comment
Kim et al. 2010 [22], Seoul, Korea.	1) 30 pts (14), 55.6 ± 8.6 y, 2) 30 pts (14), 54.7 ± 9.4 y, 3) 30 pts (14), 54.3 ± 9.1 y.	3 sex- and age-matched populations of each 30 pts: 1) pts with known T2DM, mean duration 5.3 y, mean HbA1c 7.1, 2) pts with impaired glucose tolerance (IGT), 3) pts with normal glucose tolerance (NGT).	FDG	Carotids	To examine the severity of vascular inflammation in healthy individuals without hyperlipidemia but with elevated high-sensitivity C-reactive protein (hsCRP).	TBRmean and TBRmax Carotid intima media thickness (CIMT) by ultrasound.	Subjects with high hsCRP (≥2 mg/L) and low-density lipoprotein cholesterol (LDL-C) (<130 mg/dL) levels had a significantly higher TBRmax than pts with low hsCRP (<2 mg/L) and low LDL-C levels (<130 mg/dL) (1.29±0.13, 1.12±0.10, and 1.16±0.05, respectively), even though there were **no significant differences in the CIMT**. TBRmax had strongest pos. correlation with hsCRP level among various cardiovascular risk factors (r=0.68, P<0.01). Other inflammatory markers such as lipoprotein-associated phospholipase A(2) or monocyte chemoattractant protein-1 were not associated with TBR values. hsCRP and diastolic blood pressure were independent factors for TBRmax, whereas age, diastolic blood pressure, and LDL-C determined CIMT. **Conclusion:** Vascular inflammation measured by FDG PET was increased in healthy individuals without hyperlipidemia but with elevated hsCRP.	Claims: 1) T2DM is associated with an increased risk of atherosclerotic cardiovascular disease. 2) Vascular inflammation is a key factor in both the pathogenesis and outcome of atherosclerosis. No or mainly indirect references. Questionable conclusion in that SUVmean and TBRmean values were **not higher** in IGT pts than NGT pts.
Silvola et al. 2011 [23] Turku, Finland & Munich, Germany.	LDLR−/−ApoB100/100 mice: 3 – 3 – 9 at 4, 6 and 12-17 months; IGF-II/LDLR−/−ApoB100/100 mice: 2 -3 -8 at 4, 6, 12-15 months C57BL/6N mice: 6 at 7-14 mo.	Atherosclerotic low-density lipoprotein receptor deficient mice and atherosclerotic and T2DM mice overexpressing insulin-like growth factor II compared with C57BL/6N mice on normal chow.	FDG	Aorta	Investigate effects of age, duration of a high-fat diet, and T2DM on atherosclerotic plaque development and uptake of FDG in 2 mouse models. The mice were studied at 4, 6, and 12 months of age and older after varying durations of high-fat diet.	Plaque size (intima-to-media ratio), macrophage density (Mac-3 staining), and plaque uptake of 18F-FDG were studied by means of in vivo positron emission tomography/computed tomography by ex vivo autoradiography and by histological and immunohistochemical methods.	From the ages of 4 to 6 months and 12 months and older, plaque size increased and the macrophage density decreased. Compared with controls, PET showed increased aortic FDG uptake at 4 and 6 months, but not at 12 months and older. Autoradiography showed focal FDG uptake in plaques at all time points (average plaque-to-normal vessel wall ratio: 2.4 ± 0.4, p < 0.001) with highest uptake in plaques with high macrophage density. **No differences in the plaque size, macrophage density, or uptake of FDG between the two types of mice at any time point.** Conclusions: The 6-month-old LDLR−/−ApoB100/100 and IGF-II/LDLR−/−ApoB100/100 mice demonstrated highly inflamed, large, and extensive atherosclerotic plaques after 4 months of a high-fat diet, presenting a suitable model for studying the imaging of atherosclerotic plaque inflammation with 18F-FDG. The presence of T2DM did not confound evaluation of plaque inflammation with 18F-FDG.	Numbers given are from mice studied with FDG-PET. No difference between non-diabetic and diabetic mice!

First author, time [Ref. #] Site	Study subjects, n (females) Age in years Mean±SD or range	Material	Tracer	Arterial segment	Purpose	Quantification	Main findings	Comment
Yang et al. 2012 [24] Seoul, Korea.	41 (21) T2DM pts, 56.8 ± 9.2 y and 41 (21) healthy controls, 53.3 ± 9.2 y.	T2DM duration not stated, mean HbA1c level was 6.9% [6.5–7.4] in the T2DM pts.	FDG	Right carotid artery	Soluble forms of RAGE (sRAGE), including the splice variant endogenous secretory RAGE (esRAGE), may neutralize AGE-RAGE mediated vascular damage by acting as a decoy. Association btw. serum sRAGE, esRAGE levels and vascular inflammation by PET.	TBR. In addition, relationship with CIMT, estimated GFR (eGFR), and other cardiovascular risk factors was evaluated.	Both TBRmean and TBRmax were significantly higher (+62% and +94%, respectively) in T2DM pts (mean TBR 1.08 [1.04–1.12] vs. 1.76 [1.60–1.99]; maximum TBR 1.15 [1.09–1.28] vs. 2.23 [2.14–2.38], all P < 0.001) compared to healthy subjects. Circulating sRAGE and esRAGE concentrations tended to be lower in the diabetic group, but did not reach statistical significant level (P = 0.063, P = 0.069, respectively). After adjusting for age and gender, sRAGE levels were significantly negatively correlated (large scatter) with both mean and maximum TBR values, but not with CIMT values. Maximum TBR values were independently associated with sRAGE levels in addition to HbA1c and eGFR.	Claims: "The receptor for advanced glycation end-products (RAGE) and its diverse ligands play a pivotal role in the development of cardiovascular disease." "Atherosclerosis is now commonly described as an inflammatory disease."
Brucerius et al. 2012 [25] New York, USA; Maastricht, the Netherlands, Cambridge, UK, Madrid, Spain.	43 (10) T2DM pts 61.2 ± 10.0 vs. 91 (28) non-DM pts 59.6 ± 10.2 y	Non-insulin dependent T2DM pts, mean DM duration of 7.25 y, compared to non-DM pts. All selected to have known or suspected CVD. 86% of T2DM pts and 74% of controls were on statins.	FDG	Common carotid arteries	Impact of non-insulin dependent T2DM on carotid wall FDG uptake in patients with documented or suspected cardiovascular disease.	Uncorrected SUVmax and TBRmax and glucose corrected values (suffix "gluc") applying a formula for correction of blood glucose suggested in the 2010 EANM guidelines for FDG-PET tumor imaging.	Results: The study demonstrated a significant association between diabetes and FDG uptake in the arterial wall (diabetes (mean)SUV(gluc) β = 0.324, (mean)TBR(gluc) β = 0.317, and SHS(gluc) β = 0.298; for all, p < 0.0001). In addition, in diabetic patients, both body mass index ≥ 30 kg/m(2) ((mean)SUV(gluc) β = 0.4, (mean)TBR(gluc) β = 0.357, and SHS(gluc) β = 0.388; for all, p < 0.015) and smoking ((mean)TBR(gluc), β = 0.312; SHS(gluc), β = 0.324; for all, p < 0.04) were significantly associated with FDG uptake. Conclusions: Type 2 diabetes was significantly associated with carotid wall FDG uptake in patients with known or suspected cardiovascular disease. In diabetic patients, obesity and smoking add to the risk of increased FDG uptake values.	Uncorrected mean SUVmax and mean TBRmax and single hottest segment = slice (SHS) SUVmax were all <u>lower</u> in T2DM subjects, but higher after corrections for blood glucose level!
Tarkia et al. 2015 [26] Turku, Finland.	10 pigs weighing 111 – 130 kg	Farm pigs with diabetes caused by streptozotocin injections (50 mkg/kg for 3 days) examined after 6 months of high-fat diet.	FDG	Coronary arteries	To study the uptake of FDG to various stages of coronary plaques in a pig model.	Dual-gated cardiac PET/CT. Harvested coronary segments (n=33) were examined *ex vivo* for radioactivity and with autoradiography.	Intimal thickening was observed in 16 segments and atheroma type plaques in 10 segments. Compared with the normal vessel wall, ARG showed 1.7±0.7 times higher [FDG accumulation in the intimal thickening and 4.1±2.3 times higher in the atheromas (P = 0.004 and P = 0.003, respectively). *Ex vivo* mean vessel-to-blood ratio was higher in segments with atheroma than those without atherosclerosis (2.6±1.2 vs. 1.3±0.7, P = 0.04). *In vivo* PET showed highest TBRmax of 2.7. However, TBRmax was not significantly different in	Interesting with a large animal model.

First author, time [Ref. #] Site	Study subjects, n (females) Age in years Mean±SD or range	Material	Tracer	Arterial segment	Purpose	Quantification	Main findings	Comment
							segments without atherosclerosis (1.1±0.5) and either intimal thickening (1.2±0.4, P = 1.0) or atheroma (1.6±0.6, P = 0.4). Thus, increased FDG uptake was present in coronary atherosclerotic lesions, but not detectable by in vivo PET in these early stage lesions. **Further studies are needed to clarify whether visible FDG uptake in coronary arteries represents more advanced, highly inflamed plaques.**	
de Boer et al. 2016 [27] Groningen, Amersfoort, Enschede, the Netherlands.	44 (17) 63 y (interquartile range 54-66)	Pts without CVD and anti-DM medication. Mean diabetes duration 1.0 (0.0 – 3.5) y, 24 pts (54.4%) on statins. RELEASE TRIAL1	FDG	Carotids, asc, arch, desc thoracic + abd. aorta, iliac + femoral art.	Relationship btw subclinical arterial inflammation assessed by FDG PET/CT and arterial stiffness in pts with early T2DM.	TBRmax corrected for glucose level. Central SBP (cSBP), carotid-femoral pulse wave velocity (PWV), and augmentation index (AIx).	The corrected meanTBR**max** was significantly associated with PWV (R = 0.47, P = 0.001) and cSBP (R = 0.45, P = 0.003) but not with AIx. TBRmax of each separate segment was also significantly associated with PWV and cSBP. In a multiple linear regression model including age, sex, BMI, hemoglobin A1c (HbA1c), hs-CRP, cholesterol, cSBP, and PWV, PWV was the strongest determinant of meanTBRmax. Note: TBRmax lowest in carotids, highest in the aorta (desc and abd higher than asc + arch) and intermediate in iliac + femoral arteries.	Claim: T2DM is accompanied by premature atherosclerosis and arterial stiffness. Strange with this association btw FDG uptake and PWV. Does it **reflect more chronic arterial inflammation?**
Honda et al. 2016 [28] Kurume, Japan	145 (50) 61.8±9.5 y Only 19 (13.1%) had T2DM	Pts who underwent a risk screening test for CVD	FDG	Carotids	We assessed the relationship between endothelial function and vascular inflammation evaluated by FDG-PET/CT.	120 min PET/CT acquisition to reduce blood background. TBRmax uncorrected for blood glucose. Endothelial function by flow-mediated dilation (FMD) of the brachial artery.	We investigated whether absolute changes from baseline of %FMD after antihypertensive treatment for 6 months (Δ%FMD) were correlated with those of TBR in 33 drug-naive patients with essential hypertension. Multiple logistic regression analysis revealed that age (odds ratio, 1.767 for 10-year increase), male sex (odds ratio, 0.434), low-density lipoprotein-cholesterol (odds ratio, 1.630 for 26-mg/dL increase), and TBR values (odds ratio, 1.759 for 0.2 increase) **but not diabetes** were independently associated with %FMD in the 145 patients. There was an inverse correlation between Δ%FMD and ΔTBR; ΔTBR was a sole independent associate of Δ%FMD in hypertensive patients (r=−0.558; P<0.001). The 19 T2DM pts mean TBRmax and mean HbA1c values were within normal ranges, **but HbA1c values correlated with decreased %FMD.**	Claim: Endothelial dysfunction is an initial step in atherosclerotic cardiovascular disease. Ross R. Nature 1993;362:801-9. FDG PET/CT was one of the independent correlates of decreased %FMD, suggesting the association of vascular inflammation with endothelial dysfunction in humans.
Hellberg et al. 2016 [29]		Same mice models as Silvola et al. (6): low-	FDG + fluoro-methyl-choline	Aorta	To evaluate arterial choline uptake and its relationship to	Distribution kinetics of FMCH assessed in vivo by	The aortas of all hypercholesterolemic mice showed large, macrophage-rich atherosclerotic plaques. The plaque burden and densities of macrophage subtypes were similar in	Primarily a FMCH study, but included due to FDG results showing

First author, time [Ref. #] Site	Study subjects, n (females) Age in years Mean±SD or range	Material	Tracer	Arterial segment	Purpose	Quantification	Main findings	Comment
Turku, Finland.		density lipoprotein-receptor deficient mice expressing only apolipoprotein B100, with or without T2DM caused by pancreatic overexpression of insulin-like growth factor II (IGF-II/LDLR$^{-/-}$ ApoB$^{100/100}$ and LDLR$^{-/}$ ApoB$^{100/100}$).	(FMCH)		atherosclerotic inflammation in diabetic and non-diabetic hypercholesterolemic mice.	PET. Aortic uptakes of FMCH and FDG assessed ex vivo by gamma counting and autoradiography of tissue sections. FMCH uptake in atherosclerotic plaques compared with macrophage infiltration and plasma levels of cytokines and metabolic markers.	diabetic and non-diabetic animals. The blood clearance of FMCH was rapid. Both the absolute FMCH uptake in the aorta and the aorta-to-blood uptake ratio were higher in diabetic than in non-diabetic mice. In autoradiography, the highest FMCH uptake co-localized with macrophage-rich atherosclerotic plaques. FMCH uptake in plaques correlated with levels of total cholesterol, insulin, C-peptide and leptin. In comparison with FDG, FMCH provided similar or higher plaque-to-background ratios in diabetic mice.<br>Aortic FDG uptake was higher than aortic FCHM uptake in both diabetic (4.7 vs. 2.9) and nondiabetic mice (5.0 vs. 1.5) and (as seen) similar in diabetic and nondiabetic mice.<br>.<br><br>Note figures.	similar uptake in diabetic and nondiabetic mice.

Abbreviations: ApoE-/- = apolipoprotein E knockout; BMI = body mass index; CAD = coronary artery disease; CIMT = Carotid intima media thickness; cSBP = central systolic blood pressure; CT = computed tomography; CV = cardiovascular; CVD = cardiovascular disease; DM = diabetes mellitus; EANM = European Association of Nuclear Medicine; eGFR = estimated glomerula filtration rate; esRage = endogenous secretory RAGE; FDG = 18F-fluorodeoxyglucose; FMCH = fluoro-methyl-choline; FMD = flow-mediated dilation; HbA1c = hemoglobin A1c; hsCRP = high-sensitivity C-reactive protein; IGT = impaired glucose tolerance; LDL-C = low-density lipoprotein cholesterol; NGT = normal glucose tolerance; pts = patients; PWV = pulse wave velocity; sRage = soluble forms of RAGE; SHS = Single hot segment; SUV = standardized uptake value; T2DM = type 2 diabetes mellitus; TBR = target-to-background ratio; TBRglu = glucose-corrected TBRmax; Y = years.

Table S2: Jan 2000 – Mar 2022 Studies on Early Detection and Prevalence of Arterial FDG or NaF Uptake

First author, time [Ref. #] Site	Patients, n (females) Age in years Mean±SD or range	Material	Tracer	Arterial segment	Purpose	Quantification	Main findings	Comment
Yun et al. 2001 [30] Philadelphia, US.	Total 156 (86) pts 10-96 y. Group I: 23 pts w. zero risk factors, Group II: 133 pts with ≥ 1 risk factor. Only 13 DM pts.	Pts referred for various indications. History of atherogenic risk in all pts. Diabetes meant diagnosis made by a physician, type and duration of DM not stated.	FDG	Abdominal aorta, iliac and proximal femoral arteries.	Frequency of arterial FDG uptake in relation to risk factors in a patients without (Group I) and with ≥ 1 risk factor (Group II).	Visual evaluation only.	Visible arterial FDG uptake was in general more frequent in all three arteries in group II vs. group I: Femoral 22% vs. 70%; Illiac: 30% vs 54%; Abd. aorta: 35% vs. 53%. In the 13 pts with unspecified DM, 9 (69%) had aortic, 9 had iliac (69%) and 12 (92%) had femoral artery FDG uptake. Age and hypercholesterolemia showed significant correlation with FDG uptake in all three arteries, whereas remaining risk factors (hypertension, obesity, DM, cigarette smoking) did not – however, the study was not powered to state that. Thus, it was stated that DM showed "a correlation with arterial FDG uptake only for the proximal femoral arteries".	First study reporting arterial FDG uptake in DM patients. Aimed to study frequency of FDG uptake in large arteries in relation to atherogenic risk factors. Since one of these is DM, the study holds data on prevalence of FDG uptake in DM.
Tahara et al. 2012 [31] Kurume, Japan.	Study 1: 275 (86) 61.2 ± 8.8 y and Study 2: 18 (8) pts with IGT or T2DM 65.8 ± 8.2 y	(1) 275 outpatients with mean fasting blood glucose 103.2 – 107.2 mg/dL and HbA1c 5.91 – 6.0 % and (2) 18 pts with mean fasting blood glucose 134.7 mg/dL at base and 119.4 at follow-up and HbA1c 6.87 and 6.45%.	FDG	Carotids	(1) Relation btw serum advanced glycation end products (AGEs) and vascular inflammation. (2) Are changes in AGE level after treatment with oral hypoglycemia agents (OHAs) correlated with those of TBR in 18 pts whose AGE value was >14.2 units/mL (mean ± 2 SD).	TBRmax uncurrected for prescan glucose level. CIMT	(1): Mean serum AGE level and carotid TBRmax values were 9.15 ± 2.53 and 1.43 ± 0.22 units/mL, respectively. TBR was independently correlated with AGEs (P < 0.001), carotid intima-media thickness (P < 0.01), and BMI (P < 0.02). When age- and sex-adjusted AGE values stratified by TBR tertiles were compared using ANCOVA, a significant trend was observed (P < 0.01). (2) AGE and HbA1c levels were reduced by pioglitazone but not glimepride and AGE levels after OHA treatment were positively (r = 0.50, P < 0.05) correlated with the change in TBR value, which however was not reported in numbers, but ΔTBRmax appear to change from about -03 to +0.8. Conclusions: Serum AGE level is independently associated with vascular inflammation evaluated by FDG-PET, suggesting that circulating AGE may be a biomarker that could reflect vascular inflammation within an area of atherosclerosis.	TBR values in Study 2 are not given. The study does in fact not tell anything for sure with regard to FDG uptake in prediabetic and a few patients with IGT or T2DM except that AGE level is associated with arterial inflammation by FDG PET/CT and that pioglitazone reduce HbA1c
Janssen et al. 2013 [32] Hamburg, Germany.	409 (233) 63.5±12.6 y 44 of which with unspecified DM.	Oncologic pts.	NaF	Femoral arteries	Can NaF PET/CT visualize and quantify diffuse mineral deposition in the femoral arteries? NaF uptake with CV RFs and calcified plaque burden (CPB).	TBRmax And Calcified plaque burden (CPB) = plaques with >130 HUs	"Linear" NaF uptake (PET+) in 159/409 (39%) of pts: (1) provides measure of diffuse mineral deposition, (2) demonstrates significant correlation with cardiovascular RFs and CPB, and (3) accumulate more frequently in patients with a high-risk profile for CV events. CT+ in 340/409 (83%); PET-/CT- in 56/409 (14%) pts. Max calcification in abd. aorta. About DM: of 44/409 pts with unspecified DM, 43 were CT+, 28 were NaF+, while 1 was CT-/NaF- Or in other words, the study doesn't tell very much exact about DM pts.	Diffuse and TBRmax values are incommensurable measures. The 'linear' uptake is a sort average TBRmax generated from 4 separate ROIs in each femoral artery. Nothing about calc/NaF overlap.

First author, time [Ref. #] Site	Patients, n (females) Age in years Mean±SD or range	Material	Tracer	Arterial segment	Purpose	Quantification	Main findings	Comment
Bernelot Moens et al. 2016 [33]	11 (4) controls 63±3 y; 34 (13) PAD pts 64±7 y; of which: 1) 11 (4) 2) 11 (5) 3) 12 (4).	3 random sample of PAD pts 1) PAD only 2) PAD+T2DM on oral therapy (NIDDM) 3) PAD+T2DM on combined oral/insulin (IDDM) therapy.	FDG	Carotid<br><br>TBRmax and TBRmean	The cumulative impact of PAD and type 2 diabetes on carotid arterial wall inflammation. As recent data suggest a detrimental role of exogenous insulin on cardiovascular disease, we also included a group of insulin users.	Glucose corrected (a.m. Bucerius JACC 2012) TBRmean and TBRmax calculated for both carotids as the mean of three adjoining arterial segments with highest TBR to yield most diseased segment (TBRmds).	Carotid FDG uptake compared to uptake in controls<br>1) PAD pts without DM 32% higher<br>2+3) All PAD + T2DM pts 95% higher, among these had<br>2) PAD-NIDDM pts 39% higher and<br>3) PAD-IDDM pts 122% higher despite comparable PAD severity (Fontaine stages), BMI and CRP. Multivariate regression analysis showed that Hba1c and plasma insulin levels, but not dose of exogenous insulin, correlated with TBR.<br>Conclusions: Concurrent DM significantly augments carotid arterial wall inflammation in PAD patients. Further increase in those requiring insulin, which was associated with diabetes severity, rather than use of exogenous insulin.	Nice and convincing study, providing solid and specific new knowledge about NaF uptake in DM pts.<br>Nice figures.<br>PAD defined as ankle-brachial index <0.9 and/or decrease >0.15 after treadmill test.
Bural et al. 2017 [34] Philadalphia, USA	55 (26) insulin dependent DM pts 61.5 ± 10 y and 56 (11) controls without DM and other CV RFs 61.2 ± 10 y.	110 subjects who underwent FDG PET/CT imaging for oncological diseases retrospectively studied; 55 were diabetics (blood glucose on time of injection 142 mg/dL) on insulin and 55 were age-matched controls (blood glucose 97.	FDG	Four segments (asc, arch, dsc, abd) of aorta and common iliac arteries and femoral arteries were measured.	Compare the inflammatory and macroscopic CT calcification (CTC) processes of atherosclerosis in the aortic segments and large arteries of subjects with insulin dependent DM (IDDM) to those of age-matched controls via FDG PET/CT.	Average SUVmax and SUVmean without blood glucose normalization.<br>Presence or absence of macroscopic calcification on CT images for each arterial segment was also noted	Average SUVmax and SUVmean were statistically significantly greater in subjects with IDDM compared to controls in all arterial segments (P≤0.001): Average SUVmax and SUVmean of all arterial segments were 27% and 20% higher, respectively, in IDDM pts and CTC was 27% higher.<br>Presence of calcification on CT was more frequently encountered in 6 of the 8 segments in subjects with IDDM, and there was statistically significantly difference for the descending aorta and abdominal aorta.<br>CTC highest in arc, abd and iliac in both IDDM and ctr.<br>Same pattern for FDG uptake.<br>Conclusion: inflammatory component of atherosclerosis more severe in all aortic segments in subjects with IDDM compared to those of controls. Presence of macroscopic calcification also detected to be more frequently encountered in the descending thoracic and abdominal aorta in subjects with IDDM.	Compare with Brucerius [8], who used glucose normalization: any differences?<br><br>Does these findings signal more or accelerated atherosclerosis in-IDDM?<br><br>Nice Fig. 1 showing lack of co localization of FDG uptake and vascular calcification indicating different stages in the atherosclerotic process with reference to Dunphy MP. JNM 2005;46:1278-84.

First author, time [Ref. #] Site	Patients, n (females) Age in years Mean±SD or range	Material	Tracer	Arterial segment	Purpose	Quantification	Main findings	Comment
Raggi et al. Feb 2019 [35] Edmonton Canada.	88 (31) 54.0±14.0 48 T2DM & 40 T1DM	Consecutive, well-controlled ambulatory pts with long lasting DM (median 15 y)– all asymptomatic for CVD. Mean HgbA1c 7.9%. Prospective.	NaF	Coronary arteries.	Prevalence of increased NaF uptake in pts who are usually considered at high risk of CV events by harboring potentially vulnerable coronary plaques.	TBR = SUVmax (coronary) / SUVmean LV blood pool; High TBR defined as ≥ 1.5.	Low prevalence of high coronary NaF uptake in that TBR ≥ 1.5 in only 13 pts (15%). 4 of these had no colocalized CAC, the 3 had high NaF in left main trunk without CAC in same vessel, but in other vessels. TBR associated with male sex, estimated GFR, and total coronary artery score by CT.<br>TBR > median associated with male sex and statin use.<br>No follow-up data. In fact, these data do not tell anything about CV risk, they indicate the<br>Compared to FDG uptake in DM, the frequency of pts with high NaF uptake was surprisingly low – suggesting slow atherosclerosis progression in well controlled DM or at least on little active calcification!!	According to authors: growing notion that DM pts may not be at high risk of CV events.

Abbreviations: AGEs = advanced glycation end products; BMI = bone mineral index; CAD = coronary artery disease; CIMT = Carotid intima media thickness; CPB = calcified plaque burden; CT = computed tomography; CTC = CT-calcification; CV = cardiovascular; CVD = cardiovascular disease; DM = diabetes mellitus; esRage = endogenous secretory RAGE; FDG = 18F-fluorodeoxyglucose; FMD = flow-mediated dilation; FRS = Framingham Risk Score; HbA1c = hemoglobin A1c; hsCRP = high-sensitivity C-reactive protein; IDDM = insulin dependent diabetes mellitus; IGT = impaired glucose tolerance; NaF = 18F-sodium fluoride; NIDDM = Non-insulin dependent DM; OHAs = oral hyperglycemic agents; PAD = peripheral arterial disease; pts = patients; PWV = pulse wave velocity; RF = risk factor; sRage = soluble forms of RAGE; SUV = standardized uptake value; T2DM = type 2 diabetes mellitus; TBR = target-to-background ratio; Y = years.

Table S3: Jan 2000 – Mar 2022 Studies on Cardiovascular Risk in DM

First author, time [Ref. #] Site	Patients, n (females) Age in years Mean±SD or range	Material	Tracer	Arterial segment	Purpose	Quantification	Main Findings	Comments
Strobel et al. 2013 Dec [36] Munich, Germany	315 (192) pts 57.8 ± 13.7 y	Consecutive pts PET/CT scanned for non-CV indications like malignant tumor. No. of pts with documented CV RFs: 111 Age ≥65 y 123 males, 15 DM 62 hyperlipidemia 76 hypertension 38 BMI ≥ 30, 32 current smoker.	FDG	Thoracic and abdominal aorta, both common carotids and both iliac arteries.	Effect of age, gender and CV RFs on vessel wall inflammation and the calcified plaque burden in different vascular beds as assessed by PET/CT.	Blood glucose corrected TBRmax and calcified plaque score (CPS, grade 0-4)	In thoracic aorta TBR was independently associated with age ≥65 years and male gender, CPS was independently associated with age ≥65 years, male gender, hypertension and **diabetes**. In the abdominal aorta, TBR was independently associated with age ≥65 years and male gender, CPS with age ≥65 years, **diabetes** and smoking. Independent associations in the carotid arteries were found for age ≥65 years, male gender and BMI ≥ 30 in TBR and for age ≥65 and **diabetes** in CPS. In the iliac arteries, TBR was independently associated with age ≥65, and CPS with age ≥65, male gender, hypertension, **diabetes** and smoking. Thus, impact of CV RFs on vessel wall inflammation and calcified plaque burden **differs across vascular territories. Overall, CPS was more closely associated with cardiovascular risk factors compared to TBR, which was not associated with DM in any of the 4 vascular beds.**	Claim: Is it a relevant scientific question to look for differences in association between CV RFs and FDG uptake vs. CT calcification in different vascular beds. Perhaps yes. Pts with prior or ongoing steroid medication or serious inflammation were not included.
Lee et al. 2014 [37] Suwon, Korea.	290 (132) 54.85± 8.7 y Fasting glucose 101.1±23.6 mg/dL; 92 (32.1%) had MetS	Adults who underwent FDG PET/CT as part of general health screens. Metabolic syndrome (MetS) diagnosed based on AHA criteria.	FDG	Carotids	Relation between carotid artery FDG uptake and Framingham risk score (FRS) and evaluated the possible role of FDG uptake in terms of risk stratification of asymptomatic adults.	Glucose corrected TBRmax here called "TBRglu". "High" TBRglu was arbitrarily defined as a value ≥ 1.5 corresponding to the 75th percentile in all 290 subjects.	Corrected TBRmax was 15% higher in MetS pts than pts without (1.5 vs. 1.3, p<0.001). Carotid artery FDG uptake was significantly associated with RFs. Triglyceride levels, **diabetes**, and MetS were independent determinants of high TBRglu. MetS subjects with high carotid FDG uptake had significantly higher levels of high sensitivity C-reactive protein (hsCRP). In both subjects without and with MetS, FRSs were significantly elevated in those exhibiting high instead of low carotid artery FDG uptake (13.1 ± 7.0 vs. 8.2 ± 7.4 and 21.8 ± 16.0 vs. 13.5 ± 11.9, respectively).	Here there was an association between carotid FDG uptake and FRS in pts with MetS, whereas in the study by Strobel et al. no association with CV RFs was found in DM pts. Difficult to explain.
Takx et al. 2020 [38] Utrecht & Amsterdam, NL.	68 (16) 69±8 y	Subjects with T2DM and known arterial disease (ankle-brachial index < 0.9).	NaF	Femoral.	Potential determinants of NaF uptake in these specific pts.	TBRmax	After correction for age and sex, that higher visual CT calcium in the femoral arteries, total cholesterol, and HbA1c were associated with higher NaF TBR.	Risk factors only.

Abbreviations: BMI = bone mineral index; CIMT = Carotid intima media thickness; CPS = calcified plaque score; CT = computed tomography; CV = cardiovascular; CVD = cardiovascular disease; DM = diabetes mellitus; FDG = 18F-fluorodeoxyglucose; FRS = Framingham Risk Score; hsCRP = high-sensitivity C-reactive protein; MetS = metabolic syndrome; NaF = 18F-sodium fluoride; pts = patients; pts = patients; RF = risk factor; SUV = standardized uptake value; T2DM = type 2 diabetes mellitus; TBR = target-to-background ratio; Y = years.

Table S4: Jan 2000 – Mar 2022 Studies on Disease Progression

First author, time [Ref. #] Site	Patients, n (females) Age in years Mean±SD or range	Material	Tracer	Arterial segment	Purpose	Quantification	Main findings	Comments
Reijrink et al. Sep 2021 [39] Groningen, The Netherlands.	10 (3)<br>63 (59-69) y	T2DM pts without glucose lowering drugs and without a severe CV history.	FDG & NaF	10 arteries in 4 segments: (1) carotids, 2) asc. + arch, (3) desc. + abd. aorta, (4) iliac + femoral arteries.	Prospective correlation between tracers over time and whether they are prospectively (FDG) and retrospectively (NaF) related to progression.	TBRmax (called $_{mean}$TBR by authors) using SUVmax of ten arteries divided by SUVmean of caval vein. CT calcification as calcified plaque (CP) score. Carotid-femoral PWV.	Baseline $_{mean}$TBR FDG was strongly correlated with five-year follow-up $_{mean}$TBR NaF (r = 0.709, P = .022). $_{mean}$TBR NaF correlated positively with ΔCPscore, CPscore at baseline, and follow-up (r = 0.845, P = .002 and r = 0.855, P = .002, respectively), but not with %change in CPscore and PWV.	Small study suggesting that initial FDG uptake is associated with arterial NaF uptake measured 5 years later and that the latter is associated with calcium score and PWV at baseline and 5 years, but not with 5-year change of these (!).
Bellinge et al. Jan 2021 [40] Perth, Western Australia.	41 (15)<br>65±7.1<br>and CCS≥10 compared with<br>10 (6)<br>61.2±8.4<br>and CCS=0	Pts with long lasting DM and with CT-proven CAD, but no history of clinical CAD. All had NaF-PET/CT and CT CCS scans at baseline and a repeat CT CCS scan after 2.8±0.5.<br>26/41 of high-risk and 4/10 of low-risk cohort received statins at baseline.	NaF	163 coronary arteries analyzed (one obscured by pacemaker lead).	Can localized coronary artery NaF uptake predict development of new CT detectable calcifications at least 2 years later?	CCS in Agatson units; NaF uptake in 2D ROIs around coronary arteries on 3 mm consecutive axial slices to get a SUVmax, which adjusted for right atrial blood activity yielded TBRmax.	The proportion of "CCS progressors" was higher among NaF positive than NaF negative arteries at baseline (86.5% vs 52.3%, p<0.001. NaF positive disease was an independent "predictor" of subsequent CCS progression (odds ratio 2.92 [95% CI 1.32-6.45], p=0.008. All subjects (15/15) with ≥ 2 NaF positive coronary arteries progressed in CCS.<br>Note: All pts had had previously undergone CT CCS screening and NaF PET/CT as baseline assessment for the effect of Vitamin-K1 and Colchicine on Vascular Calcification Activity in subjects with Diabetes Mellitus (ViKCoVaC) trial.	Baseline NaF uptake did not 'predict' development of new CT detectable coronary artery calcification in the individual patient. Shown was a statistically significant association between NaF positivity at baseline and CCS development.<br>Figures.

Abbreviations: BMI = bone mineral index; CAD = coronary artery disease; CCS = coronary calcium score; CIMT = Carotid intima media thickness; CP = calcified plaque; CT = computed tomography; CV = cardiovascular; CVD = cardiovascular disease; DM = diabetes mellitus; FDG = 18F-fluorodeoxyglucose; FRS = Framingham Risk Score; hsCRP = high-sensitivity C-reactive protein; MetS = metabolic syndrome; NaF = 18F-sodium fluoride; pts = patients; PWV = pulse wave velocity; RF = risk factor; SUV = standardized uptake value; T2DM = type 2 diabetes mellitus; TBR = target-to-background ratio; Y = years.

Table S5: Jan 2000 – Mar 2022 Studies on Therapy

First author, time [Ref. #] Siteb	Patients, n (females) Age in years Mean±SD or range	Material	Tracer	Arterial segment	Purpose	Quantification	Circumstances/finding	Comments
Mizoguchi et al. 2011 [41] Kurume, Japan.	52 (13) pts 68±8 y, with 2 PET/CTs out of 55 pts: 31 assigned to pioglitazone (15-30 mg/d) and 21 to glimepiride (0.5-4 mg/d)	Pts with impaired glucose tolerance (IGT) or T2DM with ultrasonic carotid atherosclerosis and evidence of FDG uptake in carotid plaque. Pts with fasting glucose ≥ 200 mg/dL, with insulin treatment, LV dysfunction or heart failure were not included.	FDG	Carotids + ascending Aorta	Compare effect of 4 months of treatment with pioglitazone, an insulin sensitizer, or glimepiride, an insulin secretagogue, on atherosclerotic plaque inflammation using serial FDG-PET imaging.	Plaque TBRmax as average of common carotid arteries and the ascending aorta. No correction for blood glucose, which was 121 and 111 mg/dL before and after in the pioglitazone group and 121 in glimepiride group.	The RCT study was completed in 31 pioglitazone-treated patients and 21 glimepiride-treated patients. Although both treatments reduced fasting plasma glucose and HbA1c values comparably, pioglitazone, but not glimepiride, decreased atherosclerotic plaque inflammation significantly: pioglitazone 1.46 → 1.32, p<0.01, glimepiride 1.35 → 1.41, NS). Compared with glimepiride, pioglitazone significantly increased high-density lipoprotein cholesterol level. High-sensitivity C-reactive protein was decreased by pioglitazone, whereas it was increased by glimepiride. The increase in high-density lipoprotein cholesterol level was independently associated with the attenuation of plaque inflammation.	**Nice Fig. 3 First study showing clear evidence of inflammation reducing effect in atherosclerosis!**
Nitta et al. 2013 [42] Kurume, Japan.	Pioglitazone group: 25 (6) 68.8±7.2 y; Glimepiride group: 22 (4) 67.3±9.3 y	Pts from the study above [25] screened for visual (grade 0-3) myocardial FDG uptake, leaving out patients with score ≥ 2. Of 50 randomized, 3 dropped out.	FDG	Left main trunk (LMT)	Compare effect of 4 month pioglitazone with glimepiride on coronary arterial inflammation with serial FDG PET/CT.	Samr as above. To reduce spill over from myocardium FDG PET/CT after 12-h fasting. TBRmax for the LMT.	After 16-week treatments, fasting plasma glucose and glycosylated hemoglobin values were comparably reduced in both groups. Reduction in TBRmax from baseline were significant for the pioglitazone group, whereas a small increase in the glimepiride group was not (1.39→1.26, p=0.033 and 1.45→1.54, NS) and so was changes in high-sensitivity C-reactive protein (pioglitazone vs. glimepiride group). None of the drugs affected vascular remodelling of the coronary arteries assessed by vessel diameter or calcification score. NICE FIGS.	From INTRO: Although pioglitazone significantly prevented the progression of coronary atherosclerosis and reduced the recurrence of myocardial infarction in patients with T2DM, see related references.
Zwackenberg et al. Oct 2019 [43] Utrecht, Groningen, etc., NL	35 (9) 69.1±8.4 y vs. 33 (7) 69.1±8.4 y	T2DM pts with CVD receiving 360 µg/d menaquinone-7 (MK-7) or placebo.	NaF	Femoral arteries	Placebo-controlled RCT.	NaF uptake (90 min acquisition) measured as TBRmax (femoral SUVmax /vena cava SUVmean) of right and left femoral artery.	Femoral artery uptake of NaF (90 min acquisition) measured as TBR (femoral SUVmax / vena cava SUVmean) was primary and calcification mass by CT was secondary outcome before and following 6-month treatment. TBRmax tended to increase (insignificantly) in the MK-7 group compared placebo while a similar tendency was not observed with regard to CT calcification, the progression of which was not reduced by vitamin K supplementation. MK-7 treatment significantly reduced dephosphorylated-uncarboxylated matrix Gla protein (as it should).	Vitamin K supplementation tended to increase femoral wall uptake of NaF compared to placebo and did not reduce CT calcification.

First author, time [Ref. #] Siteb	Patients, n (females) Age in years Mean±SD or range	Material	Tracer	Arterial segment	Purpose	Quantification	Circumstances/finding	Comments
VIrta et al. 2020 jul [44] Turku and Kuopio, Finland, Solna, Sweden.		Igf2/Ldlr-/-Apob$^{100/100}$ mice on high-fat diet (HFD) for 8 weeks and then allocated to receive HFD (n = 14) or HFD with added linagliptin (n = 15) for additional 12 weeks. Five mice fed a chow diet were controls.	FDG	Excised thoracic aorta (and liver).	Effects of the dipeptidyl peptidase 4 (DPP-4) inhibitor linagliptin on atherosclerotic plaque and hepatic inflammation.	At the end of the study, glucose tolerance, aortic and liver uptake of FDG measured by radioactivity counting and autoradiography.	Mice in linagliptin and HFD groups had similar fasting glucose concentrations, but linagliptin improved glucose tolerance. Aortas of linagliptin and HFD groups showed advanced atherosclerotic plaques with no difference in the mean intima-to-media ratio or number of macrophages in the plaques. Autoradiography showed **similar** FDG uptake by atherosclerotic plaques in linagliptin and HFD groups (plaque-to-wall ratio: 1.7 ± 0.25 vs. 1.6 ± 0.21; p = 0.24). In the liver, linagliptin reduced histologic inflammation score but had no effect on FDG uptake. Compared with chow diet, FDG uptake was similar in the aorta, but higher in the liver after HFD. Conclusions: Linagliptin therapy improved glucose tolerance and reduced hepatic inflammation but had no effect on plaque burden or atherosclerotic inflammation, as determined by histology and FDG uptake, in atherosclerotic mice with T2DM.	
Tahara et al. 2020 [45] Kurume, Japan.	39 (8) 68.0±8.1 y	38 type T2DM pts with carotid atherosclerosis who had already received OHAs except for pioglitazone were enrolled. Sub study of the Honda trial on endothelial dysfunction before and after antihypertensive treatment [11]	FDG	Left main trunk (LMT)	Assessment of which clinical variables predict change in LMT TBRmax after 4-month add-on therapy with oral hypoglycemic agents (OHAs).	At baseline and 4 months after add-on therapy with pioglitazone or glimepiride, all patients underwent 75 g oral glucose tolerance test, blood chemistry analysis, and FDG-PET/CT.	Fasting plasma glucose, 30-, 60-, 90-, 120-minutes postload plasma glucose, HbA1c, and LMT-TBRmax values were **significantly decreased** by add-on therapy, whereas high-density lipoprotein-cholesterol and adiponectin levels were increased. Increased serum levels of pigment epithelium-derived factor, a marker of insulin resistance and non-use of aspirin at baseline could predict the favorable response of LMT-TBRmax (reduction from 2.17 → 1.93, p=0.014, or – 12%) to add-on therapy. Moreover, Δ120-minutes postload plasma glucose and ΔPEDF were independent correlates of ΔLMT-TBR. Conclusions: 120-minutes postload plasma glucose and PEDF values may be markers and potential therapeutic targets of coronary artery inflammation in T2DM.	Nice Fig. 2.
Ripa et al. 2021 [46] Copenhagen, Denmark.	102 (16) 66.4±8.2 y; Linaglutide 51 (6) 65.9±8.6 y, Placebo 51 (10) 66.9±7.8 y	Pts with T2DM of 10-12 y duration.	FDG	Carotids + asc. aorta	We hypothesized that treatment with the GLP-1 RA liraglutide had a positive effect on vascular inflammation.	TBRmax in (1) all active segments (TBR >1.6), (2) most diseased segments, and (3) the whole vessels.	RCT of liraglutide (increases insulin release from beta cells) up to 1.8 mg or placebo once daily for 26 weeks. Change in the FDG TBRmax did not differ btw treatment groups: change from baseline to 26 weeks was −0.04 (95% CI, −0.17 to 0.08) in the liraglutide group and −0.09 (−0.19 to 0.01) in the placebo group (mean difference, 0.05 [95% CI, −0.11 to 0.21], P=0.53). Secondary analyses restricted to FDG uptake in the carotid arteries and other indices of atherosclerosis confirmed the primary result. Note: An explorative analysis of interaction between treatment group and history of CV indicated (P=0.052) possible effect in pts with history of CV disease.	Note that study of 64Cu-DOTATATE uptake showed reduction in the coronaries not seen in the placebo group.

First author, time [Ref. #] Siteb	Patients, n (females) Age in years Mean±SD or range	Material	Tracer	Arterial segment	Purpose	Quantification	Circumstances/finding	Comments
Bellinge et al. 2021 Apr 6 [47] Perth, Western Australia.	154 (53) ~65±7 y	Pts with selfreported approximatly 10-y lasting unspecified T1DM or T2DM and CT coronary calcifications.	NaF	Proximal coronary arteries and asc, arch, descending thoracic aorta.	TBRmax	Does vitamin-K1 or colchicine affect arterial calcification activity assessed by NaF PET?	The effect of Vitamin-K1 and Colchicine on Vascular Calcification Activity in subjects with Diabetes Mellitus (ViKCoVaC) trial of 3 months duration using a double-blind, placebo-controlled 2x2 factorial randomized design with four treatments groups (placebo/placebo, vitamin-K1 [10 mg/day]/placebo, colchicine [0.5 mg/day]/placebo, vitamin-K1 [10 mg/day]/ colchicine [0.5 mg/day]). Neither vitamin-K1 nor colchicine had a statistically significant effect on coronary TBRmax compared with placebo. No serious adverse effects reported with colchicine or vitamin-K1.	See also [40] above.
Bellinge et al. 2022 Jan 11 [48] Perth, Western Australia.	Same as above.	Same material as in [47]. 149 of the 154 completed baseline and follow-up studies.	NaF	Prox. coronaries and asc, arch, descending thoracic aorta.	Posthoc analysis of [47] using modified TBRmax limit for each coronary artery and aortic segment.	TBRmax upper limit of normal for each segment = mean TBRmax + 2 SDs in 10 DM subjects with zero coronary calcium.	Posthoc analysis of ViKCoVaC trial data. Now, vitamin K1 supplementation independently decreased the odds of developing new NaF PET positive lesions in the coronary arteries (OR: 0.35; 95% CI: 0.16, 0.78; P = 0.010), aorta (OR: 0.27; 95% CI: 0.08, 0.94; P = 0.040), and in both aortic and coronary arteries (OR: 0.28; 95% CI: 0.13, 0.63; P = 0.002).	See also [40] and [47] above.
Boswijk et al. 2022 [49]	See article.	Subanalysis of RCT including 8 male subjects at risk of developing T2DM in the shape of decreased insulin sensitivity.	FDG	Carotids + aorta and adipose tissue regions, spleen, and bone marrow.	Resveratrol has promising anti-inflammatory effects in vitro and in animal studies. This study aimed to investigate this effect on arterial inflammation in vivo.	TBRmax	Additional analysis of a double-blind randomized crossover trial. FDG PET/CT after 34 days of placebo and resveratrol treatment (150 mg/day). Median values with interquartile ranges. Arterial FDG uptake was non-significantly **higher** after resveratrol treatment (TBRmax all vessels 1.7 (1.6–1.7)) in comparison to placebo treatment (1.5 (1.4–1.6); p=0.050). Only in visceral adipose tissue, the increase in FDG uptake after resveratrol reached statistical significance (p=0.024). Furthermore, CRP-levels were not significantly affected by resveratrol treatment (p=0.091). Conclusions: Resveratrol failed to attenuate arterial or systemic inflammation as measured with FDG PET in subjects at risk of developing T2DM.	Subanalysis of Jan Brucerius material [25].

Abbreviations: CAD = coronary artery disease; CIMT = Carotid intima media thickness; CT = computed tomography; CV = cardiovascular; CVD = cardiovascular disease; DM = diabetes mellitus; FDG = 18F-fluorodeoxyglucose; FMD = flow-mediated dilation; HbA1c = hemoglobin A1c; HFD = high fat diet; hsCRP = high-sensitivity C-reactive protein; IDDM = insulin dependent diabetes mellitus; IGT = impaired glucose tolerance; LMT = left main trunk; LV = left ventricular; MK-7 = menaquinone-7; NaF = 18F-sodium fluoride; PAD = peripheral arterial disease; PWV = pulse wave velocity; sRage = soluble forms of RAGE; SUV = standardized uptake value; T2DM = type 2 diabetes mellitus; TBR = target-to-background ratio; Y = years.
